# Study of Diet Habits and Cognitive Function in the Chinese Middle-Aged and Elderly Population: The Association between Folic Acid, B Vitamins, Vitamin D, Coenzyme Q10 Supplementation and Cognitive Ability

**DOI:** 10.3390/nu15051243

**Published:** 2023-03-01

**Authors:** Xinting Jiang, Yihan Guo, Liang Cui, Lin Huang, Qihao Guo, Gaozhong Huang

**Affiliations:** 1 Department of VIP Clinical, Shanghai Sixth People’s Hospital Affiliated to Shanghai Jiao Tong University School of Medicine, Shanghai 200000, China; 2 Faculty of Medicine, The University of Queensland, Brisbane 4072, Australia; 3 Department of Gerontology, Shanghai Sixth People’s Hospital Affiliated to Shanghai Jiao Tong University School of Medicine, Shanghai 200000, China

**Keywords:** cognitive impairment, vitamins, B vitamins, vitamin D, coenzyme Q10, folic acid

## Abstract

A growing body of evidence suggests that vitamin supplements play a role in the prevention of cognitive decline. The objective of the present cross-sectional study was to evaluate the relationship between cognitive ability and folic acid, B vitamins, vitamin D (VD) and Coenzyme Q10 (CoQ10) supplementation. The sample consisted of 892 adults aged above 50 who were assessed for their cognitive status in the Shanghai Sixth People’s Hospital Affiliated to Shanghai Jiao Tong University School of Medicine (China) from July 2019 to January 2022. According to the degree of cognitive impairment, the subjects were divided into a normal control (NC) group, subjective cognitive decline (SCD) group, mild cognitive impairment (MCI) group and Alzheimer’s disease (AD) group. The results indicated a lower risk of AD in the daily VD-supplemented subjects with MCI compared to those who were not supplemented; a lower risk of cognitive impairment in those with normal cognitive who consumed VD, folic acid or CoQ10 on a daily basis compared those who did not; and a lower risk of cognitive impairment in subjects with normal cognitive performance who consumed B vitamin supplements, either daily or occasionally, compared to those who did not. The correlation was independent of other factors that potentially affect cognition, such as education level, age, etc. In conclusion, our findings confirmed a lower prevalence of cognitive impairment in those who took vitamins (folic acid, B vitamins, VD, CoQ10) daily. Therefore, we would recommend daily supplementation of vitamins (folic acid, B vitamins, VD, CoQ10), especially group B vitamins, as a potential preventive measure to slow cognitive decline and neurodegeneration in the elderly. However, for the elderly who have already suffered from cognitive impairment, VD supplementation may also be beneficial for their brains.

## 1. Introduction

Alzheimer’s disease (AD) is the most common form of dementia. The loss of short-term memory is a hallmark of its initial stage, followed by progressive impairment in multiple cognitive areas. With the aging of the population, the prevalence of neurodegenerative diseases related to aging is increasing. The latest epidemiological surveys in China and the United States show that the adjusted prevalence rate of mild cognitive impairment (MCI) is more than twice that of AD. In China, the total prevalence of dementia in people aged 60 and over is 6.0% (95%CI 5.8–6.3). It is estimated that there are about 15.07 million people with dementia, of which AD accounts for about 9.83 million (65.23%) [1,2].

Despite the in-depth study of pathological biomarkers of cognitive impairment in recent years, the clinical diagnosis of AD still depends on clinical phenotypic manifestations [3]. The diagnostic criteria may be challenged during the interval of several decades from the preclinical stage, with only pathological changes to the appearance of clinically identifiable symptoms in AD. The deficiency in the ability to encode and store new memories is characteristic of the initial stage of the disease called subjective cognitive decline (SCD), which is characterized by mild distraction. SCD can progress to MCI and be identified by neuropsychological tests. MCI is the earliest stage in which AD can be diagnosed, followed by gradual deterioration of cognitive and functional disorders, resulting in loss of independence and death [4]. Therefore, during the prodromal period of AD, we look forward to early identification of various protective and risk factors of AD and appropriate implementation of non-drug intervention strategies such as behavioral prevention strategies to provide a basis for delaying the progression of dementia [5].

In 2021, China published the Expert Consensus on Brain and Nutrition Intervention for Alzheimer’s Disease, which pointed out the three-level prevention of AD based on the theory of “intestinal flora-brain-intestine axis” and emphasized the importance of “early, coordinated, holistic and long-term” nutrition intervention [6]. As an important part of dietary nutrition, vitamins have a variety of functions in the central nervous system, which assist in maintaining brain health and optimal cognitive function. Supplementing various vitamins in the diet is considered a means to maintain cognitive function and even prevent Alzheimer’s disease [7]. The effects of multivitamins on cognitive decline and dementia have been widely studied. A meta-analysis found that supplementation of B-complex vitamins, especially folic acid, might play a positive role in delaying and preventing the risk of cognitive decline; ascorbic acid and a high dose of vitamin E also had positive effects on cognitive ability. With regard to vitamin D (VD) supplementation, the results observed in different trials varied widely, which led to a lack of certainty in assessing the potential benefits that VD might have on cognition [8]. In a randomized controlled trial (RCT), where 32 healthy adults aged 30 to 65 years old were given high-dose B vitamins, serum marker tests showed that high-dose B vitamins could reduce serum homocysteine (Hcy), indicating that high-dose B vitamin supplementation might effectively reduce oxidative stress and inflammation by increasing oxidative metabolism and promote myelination, cell metabolism and energy storage [9]. It was also found that folic acid (0.8 mg) and docosahexaenoic acid (DHA) (800 mg) supplementation, alone or in combination for 6 months, could reduce Amyloid β (Aβ)-related biomarkers and improve cognitive function in patients with MCI [10]. VD is involved in regulating the metabolism of calcium and phosphate in living organisms. A review reported the key role played by VD in the integrity of neurocognitive function, which led to a variety of cognitive symptoms when this integrity was compromised [11]. Another review demonstrated that evidence on the effects of vitamin and mineral supplements in MCI treatment was still limited [12]. Coenzyme Q10 (CoQ10) was found to be related to oxidative stress. The decline in cognitive ability might be attributed to a decline in antioxidant defense ability, reflected in the low plasma CoQ10 level in the elderly [13].

Overall, the aim of our study was to explore the relationship between vitamin supplements (folic acid, B vitamins, VD, CoQ10) and the cognitive level of the middle-aged and elderly. This was the first article in our series of studies on diet and cognitive function, followed by an exploration of cognitive function and daily dairy products, red wine, green tea, coffee, curry, common oil intake, dietary composition awareness and so on.

## 2. Materials and Methods

### 2.1. Population Study

This population-based cross-sectional study recruited those aged over 50 who took cognitive assessment in the Shanghai Sixth People’s Hospital Affiliated to Shanghai Jiao Tong University School of Medicine (China) from July 2019 to January 2022. The inclusion criteria involved participants who: (I) were 50 years old and above, regardless of sex; (II) had completed the standardized examination and diagnosed the degree of cognitive impairment. 

Participants were excluded if they met one of the following criteria: (I) missing clinical data or lost follow-up; (II) have suffered from depression, schizophrenia and other mental diseases; (III) were unable to cooperate with the inspection for various reasons; (IV) have suffered from various secondary cognitive disorders; (V) were unable to be grouped according to the diagnostic criteria of cognitive impairment. According to the inclusion and exclusion criteria, 892 questionnaires on eating habits were finally collected. Then, the subjects were divided into four groups according to their cognitive function, including 184 in the AD group, 296 in the MCI group, 227 in the SCD group and 185 in the NC group. 

The project was approved by the Ethics Committee of the Shanghai Sixth People’s Hospital Affiliated to Shanghai Jiao Tong University School of Medicine (China). The study was performed in accordance with the principles of the Declaration of Helsinki (approval number 2019-041). All participants provided written informed consent to participate in the study.

### 2.2. Data Collection

In this study, patients’ information was collected using dietary habit questionnaire, including: 1. Basic clinical information: demographic data (gender, age, years of education, height, weight, waistline, marital status, AD history of first-degree relatives), basic physical condition (tooth loss, one-year history of surgery), disease history (hypertension, diabetes, periodontitis, diarrhea, constipation, allergy history), drug history (metformin, antibiotics, personally recognized drugs) and personal history (smoking habits, drinking habits); 2. Vitamin supplements (folic acid, B vitamins, VD, CoQ10). The dietary habit questionnaire was filled out by family members or people familiar with the subjects’ eating habits so that the reliability of the questionnaire was not affected by the patients’ cognitive impairment. 

The questionnaire of dietary habits referred to the dietary guidelines of China Nutrition Society [14], dietary approaches to stop hypertension diet (DASH diet) [15], the Mediterranean-DASH intervention for neurodegenerative delay (MIND) [16], the Dutch healthy diet food frequency questionnaire (DHD-FFQ) that was used by Wesselman (2019) [17] to survey patients with SCD and the modified Mediterranean–Ketogenic diet published by Nagpal (2019) [18].

Among them, the questionnaire question about vitamins was on whether participants supplement folic acid/B vitamins/VD/CoQ10. The answers were recorded as: 1. Daily (for those who take the above vitamins at least once a day); 2. Occasionally (for those who take the above vitamins but take them less frequently or irregularly); and 3. Not at all (for those who basically do not take the above vitamins). Using the same method, we investigated other clinical data of the subjects.

### 2.3. Assessments

All participants in the study received in-depth neuropsychological evaluation that covered a wide range of cognitive functions.

We conducted medical history inquiry, physical examination, neuropsychological test, laboratory examination (genetic detection such as apolipoprotein E (APOE)) and imaging examination (brain MRI, Aβ-PET). Aβ-PET was examined in only 1/2 subjects, while MRI and APOE were completed in all subjects. The diagnosis of AD is based on NIA-AA clinical standard in 2011 [19]. The diagnosis of MCI is based on MCI generalized diagnostic standard [20] proposed by MCI International Working Group and Petersen standard [21]. The diagnosis of SCD is based on the standard proposed by Jessen et al. [22].

At the same time, we used Mini Mental State examination (MMSE), Montreal Cognitive Assessment—Basic (MoCA-B) [23] and The Addenbrook’s Cognitive Examination (ACE-III) [24] to evaluate the cognitive function of these subjects. All of our scales were evaluated by experienced clinical psychology staff who were trained prior to the project.

### 2.4. Data Analysis

Four groups of subjects were divided into three categories: the NC group and the SCD group in Category one; the MCI group and the AD group in Category two; the cognitive normal group (the NC group + the SCD group) and the cognitive impaired group (the MCI group + the AD group) in Category three. Chi-square test was used to compare the demographic data, basic physical condition, drug history, smoking habits, drinking habits and vitamin supplements (folic acid, B vitamins, VD, CoQ10) of the above three categories. Similarly, the continuous variables were compared by independent sample T test. The cognitive function of Category two and three was evaluated by covariance analysis, with age and education level as covariables. Classified variables are expressed by frequency (%), and continuous variables are expressed by mean ± standard deviation (M ± SD).

Multivariable logistic regression analysis was performed for the independent associations with cognitive level using the variables at *p* < 0.05 from the univariate analysis. We used different logistic regression models to examine the relationship between vitamin supplements (folic acid, B vitamins, VD, CoQ10) and different cognitive levels, taking vitamins (folic acid, B vitamins, VD, CoQ10) as a category (daily, occasionally, not at all). Model a did not control any variables. Model b controlled some variables, such as Category two controlled education, age, dieting to lose weight, diarrhea, allergy history and pro-cognitive drug use and Category three controlled education, age, sex, marriage, allergic history and pro-cognitive drug use. Model c adjusted for all variables in Model b + the supplementation of other vitamin variables according to the situation. The data were analyzed using SPSS25.0 software, and the difference was statistically significant with *p* < 0.05.

## 3. Results

According to the results of univariate analysis in Table 1, compared with the NC group, the SCD group had more women and more diarrhea. Compared with the MCI group, the AD group had shorter education years, older age, less diarrhea, less allergic history and less dieting to lose weight. Compared with the normal cognitive group, the cognitive impairment group had shorter education years, older age, more men and less allergic history. There was no significant difference in “Body Mass Index (BMI) and Waist” among the groups, suggesting that the overall nutritional status was similar. Whether the first-degree relatives suffered from AD or not was not related to the severity of cognitive impairment. As expected, the SCD, AD and cognitive impairment groups performed more poorly on all cognitive tests and took more pro-cognitive drugs compared with the corresponding groups in the three categories. The three categories did not differ with respect to disease history (periodontitis, hypertension, diabetes or constipation), drug history (metformin use, antibiotic use), smoking or alcohol history. In terms of vitamin supplementation, compared with the MCI group, the AD group consumed fewer VD supplements. Compared with the normal cognitive group, the cognitive impairment group consumed less folic acid, B vitamin, VD and CoQ10 supplements. Some of the participants answered, “Don’t answer”, so the sums of the different items do not match the total number of people in each group. However, the response rate for vitamin supplementation was high, as shown in Table 1.

Table 2 shows the results of binary logistic regression analysis. Compared with those having no VD supplement, individuals with MCI who used VD supplements on a daily basis had a lower risk of developing AD (OR = 0.551, 95%CI, 0.312–0.973, *p* = 0.040). After adjusting for education, age, dieting and weight loss, diarrhea, allergic history and the use of cognitive drugs, individuals with MCI who consumed VD either on a daily basis or occasionally had a lower risk of developing AD (OR = 0.395, 95%CI, 0.196–0.798, *p* = 0.010; OR = 0.572, 95%CI, 0.334–0.979, *p* = 0.042, respectively).

Table 3 shows the results of binary logistic regression analysis. Compared with those having no folic acid supplement, individuals with normal cognitive ability who used folic acid supplements on a daily basis had a lower risk of cognitive impairment (OR = 0.577, 95%CI, 0.369–0.903, *p* = 0.016). This relationship remained statistically significant after adjusting for education, age, sex, marriage, allergic history and the use of pro-cognitive drugs in Model b (OR = 0.570, 95%CI, 0.345–0.940, *p* = 0.028). Compared with those who having no B vitamin supplementation, individuals with normal cognitive ability who consumed B vitamins either on a daily basis or occasionally had a lower risk of cognitive impairment (OR = 0.389, 95%CI, 0.263–0.576, *p* < 0.001; OR = 0.522, 95%CI, 0.371–0.736, *p* < 0.001, respectively). This relationship remained statistically significant in Model b (OR = 0.391, 95% CI, 0.250–0.611, *p* < 0.001; OR = 0.639, 95%CI, 0.432–0.946, *p* = 0.025, respectively). After further adjustment of folic acid supplement, VD supplement and CoQ10 supplement in Model c, individuals with normal cognitive ability who used B vitamins on a daily basis had a lower risk of cognitive impairment (OR = 0.439, 95%CI, 0.257–0.750, *p* = 0.003). Compared with those having no VD supplement, individuals with normal cognitive ability who used VD supplements on a daily basis had a lower risk of cognitive impairment (OR = 0.526, 95%CI, 0.368–0.753, *p* < 0.001). This relationship remained statistically significant in Model b (OR = 0.532, 95%CI, 0.351–0.804, *p* = 0.003). Compared with those having no CoQ10 supplement, individuals with normal cognitive ability who used CoQ10 supplements on a daily basis had a lower risk of cognitive impairment (OR = 0.498, 95%CI, 0.333–0.745, *p* = 0.001). This relationship remained statistically significant in Model b (OR = 0.594, 95%CI, 0.380–0.929, *p* = 0.022).

## 4. Discussion

This cross-sectional study examined the intrinsic relationship between folic acid, B vitamins, VD and CoQ10 supplementation and cognitive abilities in both cognitively healthy and cognitively impaired older adults. It was revealed that VD supplementation may prevent the occurrence of cognitive impairment or delay the progress of cognitive impairment, while B vitamins, folic acid and CoQ10 supplementation could prevent the occurrence of cognitive impairment only, which means that this benefit is restricted to cognitively healthy older adults and does not extend to older adults with MCI. Hence, this finding questions the benefits of B vitamins, folic acid and CoQ10 supplementation once cognitive impairment is expressed through standardized neurocognitive testing. 

VD is a steroid hormone with biological activity in the form of 1.25 (OH)2 D. Humans have a combination of vitamins D2 (active product: ergocalciferol) and D3 (active product: cholecalciferol) available to them from ambient UV exposure (vitamin D3), habitual dietary intakes of VD-rich foods and vitamin supplements (both vitamins D2 and D3 are available) [25]. Therefore, it is impossible to differentiate whether the subjects take vitamins D2 or D3. VD plays an important role in proliferation and differentiation, calcium signal transduction within the brain, neuro-nutrition and neuroprotection. It may also alter nerve transmission and synaptic plasticity [26]. Animal experimental studies have found that high-dose VD supplementation in the early stage of the disease (before the AD symptoms appear) can improve cognitive performance [27], while supplementation of VD in the middle stage of AD disease could aggravate the neurodegeneration of AD [28]. In a cohort study of the population, Rai-Hua Lai et al. found that long-term supplementation of VD is likely to increase the risk of dementia in the elderly and increase mortality in patients with dementia [28]. However, the findings of Rai-Hua Lai et al. could not rule out the potential health benefits of VD supplementation for young people or preclinical AD patients. They [27,28] suggested that VD cannot change the cognitive function of the damaged brain, while our study revealed that VD may prevent the occurrence of cognitive impairment or delay the progress of cognitive impairment. The researchers proposed that the neuroprotective effect of VD might be related to a decrease in Aβ-related biomarkers [29]. A study by Shreeya S Navale et al. found that low levels of vitamin D were associated with an increased risk of dementia using genetic analysis and neuroimaging studies [30]. In the UK, researchers found that raising VD levels within a normal range (50 nml/L) can prevent 17% of dementia [30]. In view of the dose–response relationship between serum 25-hydroxyvitamin D (25 (OH)D) and cognitive impairment susceptibility, Ahmad Jayedi et al. conducted a meta-analysis of 1953 dementia and 1607 AD patients, reporting that a higher level of serum 25 (OH)D was associated with a lower risk of dementia and AD [31]. VD is important for normal brain development and function in rodents and humans, as its deficiency can affect cognition [32]. However, some studies could not find evidence of a relationship between VD level and cognitive function and, consequently, did not support that VD can reduce the incidence of cognitive impairment [33,34,35]. The effect of VD on cognition may be related to baseline cognitive status [27,28,35]. Larger studies need to be conducted to explore the cognitive effects of AD on people with different baseline levels.

B vitamins consist of thiamine, riboflavin, niacin, pantothenic acid, vitamin B6, folates, biotin and vitamin B12 [36]. In our survey, the subjects were not asked to specify the type of B vitamins they supplemented. Therefore, further exploration was still required for the optimal dosage of intake and the individual assessment of the effects of each B vitamin. B vitamin supplementation can reduce cognitive impairment [36]. Previous studies mainly focused on the relationship between vitamin B6, vitamin B12, folic acid and cognition. A study in India found that the elderly living in rural areas are at higher risk of a lack of trace elements, folic acid, vitamin B12 and VD [37], which is detrimental to human immune function and brain cognition. Prospective analysis found that adequate intake of folic acid, vitamin B6 and vitamin B12 was significantly associated with better cognitive reserve, which may be due to reduced hypermethylation of redox-related genes (NUDT15 and TXNRD1) and reduced oxidative damage [38]. A meta-analysis of 95 studies reported that early and long-term B vitamin supplementation could slow down cognitive decline, while higher folic acid intake was associated with a lower risk of developing dementia in older people without dementia [39]. An animal experiment reported that folic acid supplementation could reduce DNA damage as well as delay age-related cognitive decline and neurodegeneration in aging-accelerated mice with special treatment [40]. Hyper-homocysteinemia is an independent risk factor for AD. A recent dose–response meta-analysis, including a number of prospective cohort studies, showed that the relative risk of AD increased by 15% for every 5 μmol/L increase in blood Hcy [41]. It was speculated that its mechanism was not only related to the neurotoxicity of neurons caused by vascular endothelial damage but also related to the abnormal aggregation of tau protein in hippocampal neurons and the inhibition of methylation reaction [42]. Low levels of vitamin B12 are associated with low cognitive functioning in older adults [41]. RCTs reported that folic acid and vitamin B12 could cooperate to reduce the levels of Hcy and S-adenosylhomocysteine (SAH), increase the level of S-adenosylmethionine (SAM) and inhibit the expression of inflammatory factors, thus improving cognitive ability [43,44,45]. Hence, it is necessary to take enough folic acid every day to maintain normal cognitive function and reduce the risk of cognitive decline in the elderly. It is also suggested that combined supplementation of multiple nutrients may be more beneficial to improve cognitive function.

Recent studies have found that CoQ10 protects endothelial cells by promoting mitochondrial function, thus delaying age-related peripheral vascular senescence [46]. Previous studies [47,48] have found that high doses of CoQ10 may be beneficial to cognition, which is consistent with our research results. Amirreza Monsef et al. reported that a high dose of CoQ10 can improve the cognitive performance of aged healthy rats [47]. There are several explanations for why CoQ10 can prevent cognitive decline. In a study by Man Yang et al., CoQ10 was found to be an important auxiliary factor in the mitochondrial electron transfer chain. It can reduce the expression of APOE in the hippocampus by improving the energy deficiency and mitochondrial dysfunction induced by anesthesia in mice, thus alleviating the brain injury and cognitive impairment caused by sevoflurane [49]. Iman Fatemi et al. reported that chronic supplementation of CoQ10 has a potential protective effect on the brain after stroke and can reduce the sequelae of ischemia/cerebral perfusion injury, which may be related to the increase in brain-derived neurotrophic factor (BDNF) level and superoxide dismutase (SOD) activity in brain tissue [50]. Centenarians are in a state of chronic inflammation, increased oxidative stress reaction, increased CoQ10 binding protein Psap and decreased serum total CoQ10 level with age [48]. Consequently, supplementation of CoQ10 is likely to reduce the risk of age-related cognitive decline in the elderly.

The current study contributed to the growing body of research into the benefits of vitamin supplementation on cognitive health in later life by examining this association in both cognitively healthy and cognitively impaired older adults.

The main limitation of our study is its inability to determine the causal relationship between the relevant factors due to the observational study design. Moreover, this cross-sectional study does not control all confounding factors affecting cognition. We cannot rule out the potential impact of confounding factors on the results of observation. The estimates provided in this study should not be extended to other populations without additional research and validation. However, these findings still deserve further study to explore the relationship between vitamins and cognition.

## 5. Conclusions

In a word, using data from the Shanghai Sixth People’s Hospital Affiliated to Shanghai Jiao Tong University School of Medicine (China), we found that daily VD supplementation may prevent the occurrence of cognitive impairment or delay the progress of cognitive impairment, while daily B vitamins, folic acid and CoQ10 supplementation could prevent the occurrence of cognitive impairment only. Occasional VD supplementation may reduce the risk of AD. Occasional B vitamin supplements may also reduce the risk of cognitive impairment. Consequently, we provide evidence-based recommendations that daily supplementation of VD, folic acid, B vitamins and CoQ10 may be a potential preventive measure to slow cognitive decline and neurodegeneration in the elderly. However, for the elderly who have already suffered from cognitive impairment, VD supplementation may also be beneficial to their brains.

## Figures and Tables

**Table 1:** Description of the study sample based on cognitive status.

	Category One			Category Two			Category Three		
	NC Group	SCD Group	*p* ^a^	MCI Group	AD Group	*p* ^a^	Normal Cognitive Group	Cognitive Impairment Group	*p* ^a^
N	185	227		296	184		412	480	
Demographic Data									
Education	12.6 ± 2.9	12.6 ± 3.0	0.801	11.1 ± 3.2	9.6 ± 4.1	<0.001	12.6 ± 3.0	10.5 ± 3.6	<0.001
Age	65.1 ± 6.8	65.5 ± 7.9	0.593	67.7 ± 6.8	70.7 ± 7.5	<0.001	65.3 ± 7.4	68.9 ± 7.2	<0.001
BMI	23.5 ± 3.1	23.4 ± 3.3	0.765	23.6 ± 2.8	23.4 ± 3.1	0.295	23.5 ± 3.2	23.5 ± 2.9	0.799
Waist (cm)	85.0 ± 9.3	85.2 ± 9.2	0.862	85.6 ± 8.9	86.7 ± 9.2	0.205	85.1 ± 9.2	86.0 ± 9.0	0.171
Gender Man Female	68 (36.8) 117 (63.2)	57 (25.1) 170 (74.9)	0.011	103 (34.8) 193 (65.2)	73 (39.7) 111 (60.3)	0.281	125 (30.3) 287 (69.7)	176 (36.7) 304 (63.3)	0.046
Marital status Never married Married Widowed Divorced	2 (1.1) 171 (92.9) 4 (2.2) 7 (3.8)	3 (1.3) 214 (94.7) 3 (1.3) 6 (2.7)	0.820	2 (0.7) 171 (91.9) 15 (5.1) 7 (2.4)	0 (0.0) 171 (92.9) 10 (5.4) 3 (1.6)	0.823	5 (1.2) 385 (93.9) 7 (1.7) 13 (3.2)	2 (0.4) 442 (92.3) 25 (5.2) 10 (2.1)	0.002
First-degree relatives with AD YES NO	29 (17.0) 142 (83.0)	48 (21.4) 176 (78.6)	0.267	47 (16.1) 245 (83.9)	27 (14.9) 154 (85.1)	0.732	77 (19.5) 318 (80.5)	74 (15.6) 399 (84.4)	0.136
MMSE Scores	28.2 ± 1.5	27.8 ± 1.7	0.017	26.3 ± 0.2	16.8 ± 0.3	<0.001	27.5 ± 0.2	23.1 ± 0.2	<0.001
MoCA-B Scores	26.0 ± 2.5	24.9 ± 3.1	<0.001	21.1 ± 0.2	12.8 ± 0.3	<0.001	24.6 ± 0.2	18.7 ± 0.2	<0.001
ACE—III Scores	83.6 ± 6.8	79.7 ± 7.6	0.005	70.0 ± 1.2	49.6 ± 1.5	<0.001	79.5 ± 1.1	63.9 ± 1.0	<0.001
Basic physical condition									
Tooth loss No tooth loss >20 teeth 10–19 teeth 1–9 teeth No teeth	54 (29.5) 64 (35.0) 26 (14.2) 36 (19.7) 3 (1.6)	43 (19.5) 80 (36.4) 38 (17.3) 50 (22.7) 9 (4.1)	0.125	60 (21.1) 94 (33.0) 44 (15.4) 77 (27.0) 10 (3.5)	40 (22.3) 44 (24.6) 37 (20.7) 44 (24.6) 16 (7.8)	0.071	97 (24.1) 144 (35.7) 64 (15.9) 86 (21.3) 12 (3.0)	100 (21.6) 138 (29.7) 81 (17.5) 121 (26.1) 24 (5.2)	0.098
Chronic periodontitis NO YES	123 (66.8) 61 (33.2)	127 (57.7) 93 (42.3)	0.060	190 (65.7) 99 (34.3)	128 (70.7) 53 (29.3)	0.262	250 (61.9) 154 (38.1)	318 (67.7) 152 (32.3)	0.074
Periodontitis years <5 years 5–10 years >10 years	29 (47.5) 11 (18.0) 21 (34.4)	38 (41.3) 23 (25.0) 31 (33.7)	0.568	45 (46.4) 25 (25.8) 27 (27.8)	22 (41.5) 14 (26.4) 17 (32.1)	0.819	67 (43.8) 34 (22.2) 52 (34.0)	67 (44.7) 39 (26.0) 44 (29.3)	0.613
Dieting to lose weight YES NO	13 (7.1) 170 (92.9)	15 (6.7) 208 (93.3)	0.881	18 (6.2) 272 (93.8)	2 (1.1) 179 (98.9)	0.008	28 (6.9) 378 (93.1)	20 (4.2) 451 (95.8)	0.085
Diarrhea YES NO	46 (25.1) 137 (74.9)	82 (36.8) 141 (63.2)	0.012	86 (29.2) 209 (70.8)	38 (21.0) 143 (79.0)	0.049	128 (31.5) 278 (68.5)	124 (26.1) 352 (73.9)	0.073
Constipation 2–3 times/WKD 1 time/WKD 1 every 2–4 weeks no	16 (8.7) 10 (5.4) 15 (8.2) 143 (77.7)	30 (13.5) 15 (6.7) 26 (11.7) 152 (68.2)	0.188	30 (10.2) 20 (6.8) 31 (10.6) 212 (72.4)	13 (7.1) 15 (8.2) 13 (7.1) 141 (77.5)	0.345	46 (11.3) 25 (6.1) 41 (10.1) 295 (72.5)	43 (9.1) 35 (7.4) 44 (9.3) 353 (74.3)	0.608
Surgical history within 1 year YES NO	21 (11.5) 162 (88.5)	26 (11.5) 200 (88.5)	0.993	38 (12.8) 258 (87.2)	13 (7.2) 168 (92.8)	0.052	47 (11.5) 362 (88.5)	51 (10.7) 426 (89.3)	0.705
Hypertension YES NO	71 (38.4) 114 (61.6)	96 (42.5) 130 (57.5)	0.400	109 (37.2) 184 (62,8)	77 (41.8) 107 (58.2)	0.311	167 (40.6) 244 (59.4)	186 (39.0) 291 (61.0)	0.619
Diabetes YES NO	20 (10.8) 165 (89.2)	34 (15.0) 192 (85.0)	0.206	40 (13.7) 253 (86.2)	28 (15.2) 156 (84.8)	0.634	54 (13.1) 357 (86.9)	68 (14.3) 409 (85.7)	0.630
Allergic history YES NO	39 (21.1) 146 (78.9)	62 (27.4) 164 (72.6)	0.137	63 (21.3) 233 (78.7)	25 (13.7) 157 (86.3)	0.039	101 (24.6) 310 (75.4)	88 (18.4) 390 (81.6)	0.025
Drug history									
Metformin NO YES	160 (87.9) 22 (12.1)	200 (91.3) 19 (8.7)	0.262	252 (89.4) 30 (10.6)	160 (88.9) 20 (11.1)	0.873	360 (89.8) 41 (10.2)	412 (89.2) 50 (10.8)	0.775
Antibiotics YES NO	25 (13.5) 160 (86.5)	41 (18.1) 185 (81.9)	0.204	42 (14.2) 253 (85.8)	17 (9.3) 165 (90.7)	0.115	66 (16.1) 345 (83.9)	59 (12.4) 418 (87.6)	0.115
Cognitive drug use YES NO	3 (1.6) 182 (98.4)	12 (5.3) 214 (94.7)	0.047	24 (8.1) 271 (91.9)	56 (30.6) 127 (69.4)	<0.001	15 (3.6) 396 (96.4)	80 (16.7) 398 (83.3)	<0.001
Smoking habit									
Smoking at present YES NO	16 (43.2) 21 (56.8)	13 (52.0) 12 (48.0)	0.498	20 (40.8) 29 (59.2)	15 (39.5) 23 (60.5)	0.899	29 (46.8) 33 (53.2)	35 (40.2) 52 (59.8)	0.426
Secondhand smoke environment YES NO	151 (84.4) 28 (15.6)	167 (78.8) 45 (21.2)	0.158	222 (79.0) 59 (21.0)	143 (82.7) 30 (17.3)	0.341	318 (81.3) 73 (18.7)	365 (80.4) 89 (19.6)	0.731
Your parents smoked before you were born NO YES	116 (63.7) 66 (36.3)	130 (59.9) 87 (40.1)	0.433	184 (64.8) 100 (35.2)	93 (53.8) 80 (46.2)	0.023	246 (61.7) 153 (38.3)	277 (60.6) 180 (39.4)	0.755
Smoke or not >20/Day 10–20/Day 1–10/Day Quit smoking Not at all	4 (2.2) 3 (1.6) 10 (5.4) 20 (10.8) 148 (80.0)	1 (0.4) 5 (2.2) 7 (3.1) 12 (5.3) 202 (89.0)	0.063	4 (1.4) 7 (2.4) 12 (4.1) 28 (9.5) 244 (82.7)	3 (1.6) 1 (0.5) 9 (4.9) 23 (12.6) 147 (80.3)	0.466	5 (1.2) 8 (1.9) 17 (4.1) 32 (7.8) 350 (85.0)	7 (1.5) 8 (1.7) 21 (4.4) 51 (10.7) 391 (81.8)	0.646
Drinking habits									
Drinking frequency Not at all Occasionally 1–3 times/WKD >4 times/WKD	94 (51.1) 73 (39.7) 7 (3.8) 10 (5.4)	143 (63.0) 67 (29.5) 5 (2.2) 12 (5.3)	0.091	178 (60.1) 92 (31.1) 8 (2.7) 18 (6.1)	117 (63.9) 47 (25.7) 5 (2.7) 14 (7.7)	0.611	237 (57.7) 140 (34.1) 12 (2.9) 22 (5.4)	295 (61.6) 139 (29.0) 13 (2.7) 32 (6.7)	0.385
Vitamin Supplementation									
Folic acid Daily Occasionally Not at all	98.9% ^b^ 23 (12.6) 24 (13.1) 136 (74.3)	97.4% ^b^ 27 (12.2) 30 (13.6) 164 (74.2)	0.987	98.3% ^b^ 29 (10.0) 26 (8.9) 236 (81.1)	98.9% ^b^ 9 (4.9) 14 (7.7) 159 (87.4)	0.120	98.1% ^b^ 50 (12.4) 54 (13.4) 300 (74.3)	98.5% ^b^ 38 (8.0) 40 (8.5) 395 (83.5)	0.003
B vitamins Daily Occasionally Not at all	98.9% ^b^ 42 (23.0) 34 (18.6) 107 (58.5)	97.4% ^b^ 40 (18.1) 62 (28.1) 119 (53.8)	0.070	98.6% ^b^ 32 (11.0) 49 (16.8) 211 (72.3)	98.4% ^b^ 17 (9.4) 28 (15.5) 136 (75.1)	0.775	98.1% ^b^ 82 (20.3) 96 (23.8) 226 (55.9)	98.5% ^b^ 49 (10.4) 77 (16.3) 347 (73.4)	<0.001
Vitamin D Daily Occasionally Not at all	98.9% ^b^ 44 (24.0) 40 (21.9) 99 (54.1)	96.5% ^b^ 49 (22.4) 59 (26.9) 111 (50.7)	0.500	97.3% ^b^ 49 (17.0) 69 (24.0) 170 (59.0)	96.7% ^b^ 20 (11.2) 32 (18.0) 126 (70.8)	0.035	97.6% ^b^ 93 (23.1) 99 (24.6) 210 (52.2)	97.1% ^b^ 69 (14.8) 101 (21.7) 296 (63.5)	0.001
CoQ10 Daily Occasionally Not at all	98.9% ^b^ 34 (18.6) 19 (10.4) 130 (71.0)	96.9% ^b^ 36 (16.4) 29 (13.2) 155 (70.5)	0.624	97.3% ^b^ 32 (11.1) 31 (10.8) 225 (78.1)	97.8% ^b^ 14 (7.8) 15 (8.3) 151 (83.9)	0.305	97.8% ^b^ 70 (17.4) 48 (11.9) 285 (70.7)	97.5% ^b^ 46 (9.8) 46 (9.8) 376 (80.3)	0.002

Notes: ^a^ Continuous variables were analyzed using independent sample T test, whereas categorical variables (proportions) were analyzed using the chi-square test. ^b^ Response rate. *p*, *p* value. NC, normal control; SCD, subjective cognitive decline; MCI, mild cognitive impairment; AD, Alzheimer’s disease; BMI, Body Mass Index; MMSE, Mini Mental State examination; MoCA-B, Montreal Cognitive Assessment—Basic; ACE-III, The Addenbrook’s Cognitive Examination; WKD, Weekend; CoQ10, Coenzyme Q10.

**Table 2:** Multivariate analysis of vitamin D supplementation (as categorical variable) (comparison between the MCI group and the AD group).

	Reference Group		B	SE	Wald	df	*p*	OR	95%CI
Vitamin D									
Model a	Not at all	Everyday	−0.597	0.290	4.225	1	0.040	0.551	0.312–0.973
	Not at all	Occasionally	−0.469	0.244	3.691	1	0.055	0.626	0.388–1.010
Model b	Not at all	Everyday	−0.929	0.359	6.708	1	0.010	0.395	0.196–0.798
	Not at all	Occasionally	−0.559	0.274	4.152	1	0.042	0.572	0.334–0.979

Notes: a, basic model, no adjustment. b, adjusted for education, age, dieting to lose weight, diarrhea, allergy history, pro-cognitive drug use. MCI, mild cognitive impairment; AD, Alzheimer’s disease; B, Coefficient for the constant; SE, Standard error around the coefficient for the constant; Wald chi square statistics; df, degree of freedom for Wald chi square statistics; *p*, *p* value; OR, Exp (B); 95%CI, Confidence interval for the odds ratio with its upper and lower limits.

**Table 3:** Multivariate analysis of folic acid, B vitamins, vitamin D, CoQ10 (as categorical variable) (comparison between the normal cognitive group and the cognitive impairment group).

	Reference Group		B	SE	Wald	df	*p*	OR	95%CI
Folic Acid									
Model a	Not at all	Everyday	−0.550	0.228	5.787	1	0.016	0.577	0.369–0.903
Model b	Not at all	Everyday	−0.562	0.256	4.843	1	0.028	0.570	0.345–0.940
B vitamins									
Model a	Not at all	Everyday	−0.944	0.200	22.314	1	0.000	0.389	0.263–0.576
	Not at all	Occasionally	−0.649	0.175	13.729	1	0.000	0.522	0.371–0.736
Model b	Not at all	Everyday	−0.939	0.228	17.003	1	0.000	0.391	0.250–0.611
	Not at all	Occasionally	−0.447	0.200	5.000	1	0.025	0.639	0.432–0.946
Model c	Not at all	Everyday	−0.824	0.273	9.088	1	0.003	0.439	0.257–0.750
Vitamin D									
Model a	Not at all	Everyday	−0.642	0.183	12.336	1	0.000	0.526	0.368–0.753
Model b	Not at all	Everyday	−0.632	0.211	8.968	1	0.003	0.532	0.351–0.804
CoQ10									
Model a	Not at all	Everyday	−0.697	0.205	11.512	1	0.001	0.498	0.333–0.745
Model b	Not at all	Everyday	−0.520	0.228	5.222	1	0.022	0.594	0.380–0.929

Notes: a, basic model, no adjustment. b, adjusted for education, age, sex, marriage, allergic history, pro-cognitive drug use, c, adjusted for all variables in model b + the supplementation of folic acid, Vitamin D, CoQ10. CoQ10, Coenzyme Q10; B, Coefficient for the constant; SE, Standard error around the coefficient for the constant; Wald chi square statistics; df, degree of freedom for Wald chi square statistics; *p*, *p* value; OR, Exp(B); 95%CI, Confidence interval for the odds ratio with its upper and lower limits.

## Data Availability

The data presented in this study are available on request from the corresponding author. The data are not publicly available due to confidentiality issues.
